# Surgery

**Published:** 1904-12

**Authors:** James Swain


### SURGERY.

**Mesenteric embolism and thrombosis,** though comparatively infrequent, is of great importance to the abdominal surgeon on account of the close resemblance of the symptoms to those of ntestinal obstruction. Drs. Jackson, Porter and Quinby,[2] by an analysis of the records of 214 cases, have done much to elucidate this difficult subject.

Virchow described the affection pathologically in 1847, Litten wrote on its clinical aspects in 1875, and since then various experiments have been made by Cohnheim, Cohn, and others, demonstrating the results of the stoppage of the blood supply of the intestines.

Considering the richness of the vasular anastomoses of the intestine, it is noteworthy that closure of the mesenteric vessels does not, as a rule, call forth a competent collateral circulation, as is usually the case in other parts of the body. Various theories have been propounded to explain this fact, but perhaps the most satisfactory is that of Faber,[3] who says the infarction is due to back pressure of blood in the portal system, which overcomes and is greater than the pressure in the anastomosing vessels. The reason why the inferior mesenteric artery does not take up the work of the closed superior mesenteric is due to the fact of its being a smaller vessel, and unable therefore to assume the functions of a larger one. Further, after closure of the superior mesenteric artery, the pressure in the superior mesenteric vein necessarily sinks to *nil* immediately. The pressure in the portal system is thus lowered, making the flow from the inferior mesenteric vein easier; and it is thus easier for blood in the inferior mesenteric artery to flow by its natural channel. Whether the flow is interfered with in the inferior mesenteric artery alone, or the vein alone or both simultaneously, the results are identically the same—first necrosis, second gangrene, third hemorrhagic infarction, and fourth abscess formation.

The **pathological evidence** depends largely on the duration of the process. In most cases there is free fluid in the general

[1] *Loc. cit.* [2] *J. Am. M. Ass.*, 1904, xlii. 1469; xliii 25, 110, 183.

[3] *Deutsches Arch. f. klin. Med.*, xvi. 527.

peritoneal cavity, often blood-stained, and usually in sufficient amount to be demonstrable during life. In more than half the cases the infarcted area has a well-marked line of demarcation, but it is less marked in some cases where the collateral circulation has been partly developed. The contents of the intestine are bloody in most cases. Ulcerations of the intestinal mucosa are not uncommon. The mesentery is often thickened and œdematous, and sometimes the blood forms so considerable a tumour between its layers that it can be felt on palpation. The mesenteric glands and lymphatic tissue are often swollen. In cases of venous closure, as opposed to embolus of the mesenteric arteries, the large intestine is only seldom affected, owing, doubtless, to the more abundant anastomoses between the inferior mesenteric vein and the inferior vena cava with the hemorrhoidal vein.

As regards the **etiology,** we find that all those diseases which lead to the formation of thrombi whence emboli may arise are of direct influence as causes of mesenteric occlusion. Endocarditis, atheroma of the aorta, and arterio-sclerosis, especially of the mesenteric arteries, are of the greatest importance. In the case of venous thrombosis all conditions causing stasis in the portal system play a causative rôle. The thrombosis may be primary and ascending, or it may be secondary to a process beginning higher up—both classes occur. All intestinal changes which allow penetration of bacteria into the vessels are of importance in the causation of the primary thrombosis. Such are the severe forms of enteritis, surgical infections, phlebitis of the lower extremities, and various cachexias such as those due to cancer, malaria, sepsis, typhoid fever, and appendicitis. Secondary venous thrombosis follows cirrhosis and syphilis of the liver, pylephlebitis and processes at the liver hilum, which by pressure, or the formation of adhesions, cause portal stasis.

The **symptoms** may be divided into two groups—acute and chronic. The first group is by far the larger, and is composed of cases of sudden onset of colicky abdominal pain, followed by nausea and vomiting and diarrhœa, both of which are often bloody, or the case resembles one of obstinate intestinal obstruction of the paralytic type. Often not even flatus is passed, the abdomen rapidly becomes distended with gas, peristalsis is absent, and death occurs in a few hours or days. The second and smaller group is formed by cases of insidious onset, and chronic, sometimes remitting symptoms, by cases having no symptoms referable to the abdomen during life, and by those where spontaneous cure resulted. There appears to be no difference as regards acuteness of onset, whether the condition is one of arterial embolism or venous thrombosis, so that the commonly-accepted view that the acute cases are dependent on embolism and the chronic ones upon thrombosis is not correct. The cases are about twice as common in men as in women, and over one-half of them occur between 30 and 60 years of age.

As regards **diagnosis**, Gerhardt considers the following signs as necessary to a diagnosis of this affection:—1. The presence of a source for an embolus. 2. Copious intestinal hemorrhages, not to be explained by disease of the wall of the bowel, or by hindrance to the portal circulation. 3. A rapid and marked fall of temperature. 4. Colicky (often severe) pains in the abdomen. 5. Later, distension of the abdomen and the presence of free fluid. 6. The simultaneous or previous occurrences of embolism in other parts. 7. The occasional presence of a tumour in the abdomen due to the infiltration of the mesentery by blood. The existence of some of these signs is necessary for the making of a diagnosis, though it is rare to find them all present in any one case. The conditions most often confused with these cases of intestinal paralysis are those where the obstruction is due to a mechanical hindrance. These are intussusception, volvulus, strangulation by bands, or obstruction from gallstones or cancer. It is also necessary to exclude all those diseases which may cause blood in the stools or vomit, such as gastric or duodenal ulcer, and diseases of the heart and liver. In the great majority of cases it will be found that a diagnosis of intestinal obstruction is made, and this seems as near as we can get at present.

The **prognosis** is bad, and the mortality is given as 94 per cent. Even in the more chronic cases the outlook is unfavourable, because of the hindrance to the formation of an efficient collateral circulation.

The only rational **treatment** is that of exploratory laparotomy in every case where the patient's general condition will warrant it, and as soon as ever a probable diagnosis has been made. The gangrenous part should be resected, but so far only four cases (8 per cent.) have recovered. The authors think this high mortality may be due to faulty technique. In many cases it is impossible, even with good demarcation lines, to be sure that the gangrene has reached its limits, and therefore it seems unwise that resection should be followed by immediate anastomosis. Moreover, this procedure exposes the patient to an operation which is too long and involves too much trauma. They therefore advise that the involved gut should be brought well out of the wound, with liberal sound margins left at either end, and after resection that the open ends should be fixed in the wound well walled off with gauze tampons. If peritonitis is present a speedy flushing out of the cavity with hot saline solution is advisable, and adds but little to the danger of the operation. With both ends of the intestine thus open the distension can be relieved and the intestine watched for signs of further gangrenous involvement. This method, the details of which must vary with the preference of the individual surgeon, appears to put the patient in the best position for recovery if such be possible.

JAMES SWAIN.

